# Improving the effects of drought priming against post-anthesis drought stress in wheat (*Triticum aestivum* L.) using nitrogen

**DOI:** 10.3389/fpls.2022.965996

**Published:** 2022-08-10

**Authors:** Attiq Ullah, Zhongwei Tian, Libing Xu, Muhammad Abid, Kangqi Lei, Anab Khanzada, Muhammad Zeeshan, Chuanjiao Sun, Jinhong Yu, Tingbo Dai

**Affiliations:** ^1^Key Laboratory of Crop Physiology, Ecology and Production Management, Nanjing Agricultural University, Nanjing, China; ^2^Department of Soil Conservation, Narowal, Pakistan; ^3^Key Laboratory of Crop Cultivation and Tillage, Agricultural College of Guangxi University, Nanning, China

**Keywords:** drought priming, antioxidant, abiotic stress, photosynthesis, wheat

## Abstract

Water and nitrogen (N) deficiencies are the major limitations to crop production, particularly when they occur simultaneously. By supporting metabolism, even when tissue water capacity is lower, nitrogen and priming may reduce drought pressure on plants. Therefore, the current study investigates the impact of nitrogen and priming on wheat to minimize post-anthesis drought stress. Plant morphology, physiology, and biochemical changes were observed before, during, and after stress at the post-anthesis stage. The plants were exposed to three water levels, i.e., well watering (WW), water deficit (WD), and priming at jointing and water deficit (PJWD) at the post-anthesis stage, and two different nitrogen levels, i.e., N180 (N1) and N300 (N2). Nitrogen was applied in three splits, namely, sowing, jointing, and booting stages. The results showed that the photosynthesis of plants with N1 was significantly reduced under drought stress. Moreover, drought stress affected chlorophyll (Chl) fluorescence and water-related parameters (osmotic potential, leaf water potential, and relative water content), grain filling duration (GFD), and grain yield. In contrast, PJWD couple with high nitrogen treatment (N300 kg ha^–1^) induced the antioxidant activity of peroxidase (37.5%), superoxide dismutase (29.64%), and catalase (65.66%) in flag leaves, whereas the levels of hydrogen peroxide (H_2_O_2_) and superoxide anion radical (O_2_^–^) declined by 58.56 and 66.64%, respectively. However, during the drought period, the primed plants under high nitrogen treatment (N300 kg ha^–1^) maintained higher Chl content, leaf water potential, and lowered lipid peroxidation (61%) (related to higher activities of ascorbate peroxidase and superoxide dismutase). Plants under high nitrogen treatment (N300 kg ha^–1^) showed deferred senescence, improved GFD, and grain yield. Consequently, the research showed that high nitrogen dose (N300 kg ha^–1^) played a synergistic role in enhancing the drought tolerance effects of priming under post-anthesis drought stress in wheat.

## Introduction

Plant growth and development are greatly associated with the available resources, such as nitrogen, water, and light. Studies have shown that water and nitrogen deficiencies alter numerous morphological and physiological processes in plants (Wang et al., 2021). Water scarcity is viewed as the most limiting factor to plant development and yield (Siddiqui et al., 2015; Jabborova et al., 2021). Wheat (*Triticum aestivum* L.) is considered to be the largest cultivated cereal crop in the world and is extremely sensitive to drought stress, which regularly occurs at the post-anthesis stage and leads to significant yield losses (Cossani and Reynolds, 2012; Wang et al., 2014a). Drought stress induced during the post-anthesis stage of the wheat crop either halts grain filling or causes the grain to remain completely unfilled (Dhanda and Sethi, 2002), as grain filling is predominantly a connected source–sink relationship (Thakur et al., 2010). In cereals, photosynthesis of the flag leaf and inflorescence during post-anthesis plays a crucial role in grain development (Murchie et al., 2002). Therefore, improving drought tolerance during post-anthesis in wheat is crucial for sustaining food security in the present global climate scenario.

The recent global climate events are continuously impacting agriculture, threatening global food security. Drought stress is the primary factor affecting plant metabolism and disturbing the photosynthesis machinery (Ahmad S. et al., 2021; Nardino et al., 2022). In the process, drought stress reduces the stomata conductance to restrict water losses during evapo-transpiration and reduces the CO_2_ in the grass (Gaikwad et al., 2020), subsequently disturbing photosystem II (Fv/Fm) (Grassi and Magnani, 2005). In fact, the effects of drought on wheat crops are multilayered, such as turgor loss, osmotic imbalance, and shrinkage in cell volume, as well as reduced membrane stability and accumulation of ROS (Batra et al., 2014; Liu et al., 2015; Ahmad A. et al., 2021). Moreover, the failure of plants to scavenge ROS induces oxidative stress, which leads to membrane lipid peroxidation, protein oxidation, hormonal imbalance, and finally, cell death (Niu et al., 2013; Liu et al., 2015). Drought tolerance is a complex quantitative trait (Wu et al., 2022), and plants deploy multiple strategies to combat drought (Ahmad et al., 2019). These include osmoprotectants, enzymatic and non-enzymatic antioxidants, biomarkers, and secondary metabolism-related enzymes (Zeeshan et al., 2020; Siddiqui et al., 2021; Salam et al., 2022).

Previous studies indicated that pre-stress priming had a positive effect on plants by activating faster and more proficient defense mechanisms against the subsequent stress circumstances (Abid et al., 2016b; Wang et al., 2018). Pre-stress priming, which is known as “stress memory” (Wang et al., 2018), could increase stress tolerance (Abid et al., 2016b). Previous experiments showed that induction of drought priming at the vegetative stage and/or pre-anthesis stage enhanced heat and/or drought tolerance during the grain filling stage in *Triticum aestivum* L. (Wang et al., 2014a,b) and enhanced freezing tolerance in wheat at the jointing stage (Li et al., 2015). Similarly, high-temperature stress at the post-anthesis stage was relieved in response to drought priming in wheat, as evident by enhanced photosynthetic activity and mitigation of oxidative stress (Zhang et al., 2016). Moreover, higher net photosynthesis along with dry biomass and grain yield was observed in response to the multiple water-logging priming induced at three different growth stages in wheat (Li et al., 2011). Therefore, inducing priming at different growth stages against the corresponding stresses occurring during post-anthesis was found to be an effective tool for stress mitigation. But it should be noted that drought stress levels change during the vegetative development period of wheat, and therefore the effects of drought priming against post-anthesis drought stress require further investigation. Additionally, previous studies did not investigate the use of high nitrogen fertilizer along with pre-stress priming to enhance drought tolerance.

Nitrogen is a significant underlying part of nucleic acids, proteins, chlorophyll, rubisco, and some hormones, and can decrease drought stress damage by maintaining metabolic activities (Zhang et al., 2007; Ata-Ul-Karim et al., 2016). Abid et al. (2016a) suggested that proper nitrogen supply can improve plant drought tolerance in wheat. Studies reported that the utilization of nitrogen fertilizer relieves the negative impact of drought stress (Saneoka et al., 2004; Yang et al., 2012). Morgan (1986) suggested that nitrogen-fertilized wheat plants respond faster to drought stress by closing stomata and reducing net photosynthesis. Shabbir et al. (2016) found that a combined foliar spray of additional nitrogen affected the addition of osmoprotectants and the activities of nitrogen assimilation and antioxidant enzymes, thereby improving wheat yield and drought tolerance. Moreover, water limitation and low nitrogen supply are the main limiting factors for wheat yield. It is widely reported that both factors affect leaf–water relationship, chlorophyll fluorescence, and photosynthetic processes, thereby limiting the rate of plant development, inducing early senescence, and shortening the GFD period, thus causing limited grain weight and poor crop productivity (Madani et al., 2010; Mobasser et al., 2014). However, Li et al. (2011) revealed that under drought stress, applying nitrogen prevents different grass species from changing their physiological functions. These varying outcomes may be accredited to differences in particular environments, species characteristics, drought stress levels, nitrogen, timing, or different growth phases of the crops. Even under constant water and nitrogen levels, the response of plants to drought stress varies at various phases of crop development (Ernst et al., 2010; Shi et al., 2014). In addition, despite its significance, very few studies can completely compare the effects of different levels of drought stress on grain yield traits at different vegetative stages of wheat. Furthermore, the tandem use of high nitrogen fertilizer and priming to understand the plant ecophysiological mechanisms of drought resistance has not been explored. Therefore, an appropriate estimation of the effects of drought priming against post-anthesis drought stress using nitrogen on the physiological activities and yield attributes will provide significant insight into the cultivation of wheat under adverse conditions (Teixeira et al., 2014).

In this study, we hypothesized that nitrogen application and pre-anthesis drought priming might lessen the effects of post-anthesis drought stress in wheat. It was further hypothesized that an appropriate increase in nitrogen application in water deficit conditions would enhance the drought tolerance of the wheat crops.

## Materials and methods

### Experimental design

**FIGURE 1:**
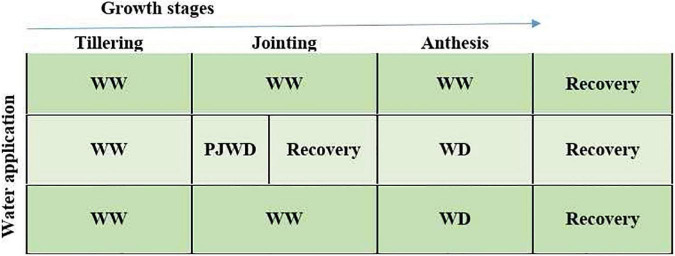
Experimental plan for the study: drought priming (P) was applied at jointing (J) by exposing the plants of two wheat cultivars (Yangmai-158 and Yangmai-22) to moderate drought stress at 55–60% field capacity (FC) for 10 days followed by re-watering. Meanwhile, non-priming plants (WW) were subjected to continued watering at 75–80% FC. At the post-anthesis stage, moderate drought stress at 55–60% FC was applied from 7 to 14 days after post-anthesis followed by re-watering. The treatments during post-anthesis drought stress were assigned as PJWD priming at jointing + post-anthesis drought stress, WD no priming at jointing + post-anthesis drought stress, and WW no priming at jointing + no post-anthesis drought stress. After priming at jointing and application of post-anthesis drought stress treatments, the plants were re-watered directly at 75%–80% FC for recuperation.

To assess the combined effects of nitrogen and pre-drought priming on the wheat plants during post-anthesis drought stress, a rain cover pot experiment was conducted at the Pailou Experimental Research Station of Nanjing Agricultural University, China (32°040 N, 118°760 E), during the growing period of 2018–2019. Plants were sown at a shallow depth of 3–5 cm in plastic pots, i.e., 25 cm high and 22 cm in diameter, and filled with 10 kg of clay loam air-dried soil (bulk density; 1.29 g cm^–3^), sieved with a 0.5-mm mesh, uniformly mixed, and placed in a shade house under natural light conditions. Meanwhile, at the time of pot filling, 0.5 g of P_2_O_5_ and 1.1 g of K_2_O per pot were mixed with soil. Two wheat varieties, i.e., Yangmai-158 (medium gluten) and Yangmai-22 (low gluten), were seeded as experimental materials. Prior to sowing, seeds were disinfected with 1% (v/v) H_2_O_2_ and then washed three times using ddH_2_O. Fourteen seeds of each variety were sown in each pot and were arranged in a completely randomized design (CRD) with a factorial arrangement. The experimental design is presented in Figure 1. At emergence, seven uniform seedlings were left after thinning in all pots for subsequent experimental measurements and analysis. The details of the atmospheric temperature of the experimental area are given in Table 1.

**TABLE 1:** Average temperatures during growing seasons of 2018–2019.

Temperature	2018		2019				
	Nov	Dec	Jan	Feb	Mar	April	May
Min. temperature (°C)	9.5	2.9	0.83	0.07	7.19	13.23	16.87
Max, temperature (°C)	17.1	8.61	7.32	7.28	16.12	22.1	26.19

### Treatments and applications

The experiment consisted of two wheat varieties, i.e., Yangmai-158 (YM-158) and Yangmai-22 (YM-22), and three water levels, i.e., well watering (WW), water deficit (WD), and priming at jointing and water deficit (PJWD) at post-anthesis stage. The experiment included two different nitrogen levels, i.e., N180 kg ha^–1^ (N1) and N300 kg ha^–1^ (N2). There were 12 treatments and 33 pots as replicates per treatment (2 variety* 3 water level* 2 nitrogen* 33 replications = 396 pots in total). Drought priming was induced at the jointing stage, and irrigation of pots was suspended until a moderate drought stress level from 55 to 60% field capacity (FC) was reached. This moderate drought stress level was maintained for 10 days as priming by compensating for water lost every day. Meanwhile, the control pots were kept on irrigation under well-watered conditions. After the completion of priming, the pots were re-watered to the level of control pots (78–80% FC) until the application ensured post-anthesis drought stress. Seven days after the anthesis appeared, plants were exposed to drought stress (at 30–40% field capacity) and re-watered to 78–80% FC till harvesting after the complication of treatment duration. The soil moisture content of each treatment was measured daily using an HH2 Moisture Meter (Delta-T Devices, Cambridge, United Kingdom). For N treatment, nitrogen fertilizer in the form of urea nitrogen [CO(NH_2_)_2_] was applied in three splits: 50% N of each level was applied at the time of sowing, another 30% N at jointing, and another 20% at booting. The quantity of water required for pot irrigation was determined using the procedure suggested by a previous study (Abid et al., 2016a).

W=Y×H×A×(F⁢C⁢1-F⁢C⁢0)

where W is the quantity of water irrigation, H is the depth of soil, A is the vicinity of pot, Y is the bulk density of the soil, FC1 is the top edge of the needed soil FC, and FC0 is the real soil FC before irrigation.

### Plant selection and trait measurement

At the post-anthesis stage, the uniform tillers/spikes from all treatments were tagged at the same time for testing and measurements. The topmost fully expended flag leaves were sampled from the three chosen pots (considered as one replication) of each treatment 1 day before the beginning of drought stress (0 DS), 5 days after drought stress (5 DS), and 5 and 10 days after re-watering (5 DRW and 10 DRW) for the determination of physiological and biochemical parameters. Plants in each pot were utilized only once per sample at each time point and then were discarded. All plant samplings and photosynthesis measurements were performed between 9:00 a.m. and 11:00 a.m. local time. The samples collected for protein assay and other related parameters were kept at –80°C until further determination.

### Osmotic potential, leaf water potential, and leaf relative water content

The flag was selected for measurement of leaf relative water content (LRWC) from each treatment in three replications as indicated by a previous study (Barrs and Weatherley, 1962). The LRWC equation is as follows:

$$L\,R\,W\,C=\frac{\left(\text{FW}-\text{DW}\right)}{\left(\text{TW}-\text{DW}\right)}\times 100$$

where FW indicates the fresh weight of leaf, TW indicates the fresh weight of turgid dipped leaves in distal water after drenching in the darkness for 24 h, and DW indicates the dry weight of leaves after dehydration at 75°C to a stable weight (oven drying).

The osmotic potential was resolved by following the technique of a previous study (Ribaut and Pilet, 1991). The novel completely enlarged leaf was set in the nitrogen fluid at a defrosted point (25°C), and the cell sap liquid was separated by compacting the leaf in a syringe. The osmotic concentration was estimated using the fume osmometer pressure (Wescor Vapor 5600, United States) and determined using the van’t Hoff condition.

The leaves were immobilized in the fixed cover of the sample holder, and pressure was applied until liquid flowed from the uncovered end of the leaf stalk. At this point, careful perusing was noted, which demonstrated that water was held inside the leaf with negative power, expressed as –MPa.

### Leaf gas exchange measurements

At the post-flowering stage, IRGA Li-COR portable photosynthesis system (LI-6400 Inc., Lincoln, NE, United States) was used to measure gas exchange measurements in the flag leaf. Photosynthesis was measured at 1,000 μmol photo m^–2^ s^–1^ at 25°C and 400 μmol mol^–1^ CO_2_ (Ca). The light saturation net CO_2_ assimilation rate (Pn) and stomatal conductivity (*gs*) were determined.

### Chlorophyll fluorescence measurements

The measurements of chlorophyll fluorescence were performed using a modulated fluorimeter (FMS2; Hansatech, King’s Lynn, Norfolk, United Kingdom). Prior to measurements, the topmost fully expanded leaf that was uniformly oriented and fully exposed to light was kept in the dark for 30 min, and then exposed to light (∼1,000 m mol photons m^–2^ s^–1^) to record the ground state fluorescence (F0) and the maximum fluorescence (Fm). At this point, the steady-state fluorescence value (Fs) and maximum fluorescence (Fm’) of the light adaptation level were measured. Next, the actinic light was turned off, and the leaf was illuminated with far-red light for 3 s to determine the minimum fluorescence in the light-adapted state (F0’). Based on these standards, according to the recommendations from Maxwell and Johnson (2000), the fluorescence was determined using the maximum efficiency of PSII (Fv/Fm) and the effective quantum yield of PSII (ΦPSII).

### Chlorophyll and soluble protein content measurements

A handheld SPAD 502 Chl meter (Soil Plant Analysis Development; Minolta, Japan) was utilized to measure the chlorophyll content (SPAD) of flag leaves of each treatment. The SPAD readings were taken from five flag leaves of each pot, averaged, and considered as one replication. Similarly, three replications were taken per treatment, and the averaged values were taken as the chlorophyll content.

To quantify the total soluble protein content, fresh leaves (0.5 g) were ground in sodium phosphate solution (50 mM, pH 7.0). The amalgam was centrifuged at 4,000 × *g* at 4°C for 10 min. In order to measure the soluble proteins, bovine serum albumin was used to normalize the sample supernatant, and the absorbance value was recorded spectroscopically at 595 nm.

### Superoxide production, malondialdehyde content, hydrogen peroxide content, and antioxidant enzyme activity

The O_2_^–^ content was estimated according to the method suggested by Sui et al. (2007). The H_2_O_2_ and malondialdehyde (MDA) contents were determined according to the method by Zheng et al. (2009). The activity of superoxide dismutase (SOD, EC 1.15.1.1) was assessed as suggested by Yang et al. (2007), and the activity of peroxidase (POD, EC 1.11.1.7) was determined according to the method by Zheng et al. (2009). The catalase activity (CAT, EC 1.11.1.6) was assayed using the method of Tan et al. (2008).

### Flag leaf area and weight, grain filling duration, and yield component determination

At the post-anthesis stage, the **LI-3000** area meter (Li-Cor. Inc., Lincoln, NE, United States) was used to measure the green flag leaf area during drought stress treatment (WD). The specific leaf weight was calculated as follows:

S⁢W=W⁢L/A⁢L

where WL is the flag leaf weight and AL is the flag leaf area.

When half of the spikes under the treatment had reached grain maturity, the grain filling duration (GFD) (the number of days from blossoming to grain development) was recorded. At maturity, three pots were haphazardly chosen from every treatment to ascertain the number of tillers with spikes, the number of grains/spikes, spike length, the weight of 1,000 grains, grain yield/pot, plant biomass, and drought index (DI). The DI was determined as the proportion of grain yield under drought stress (YD) and grain yield under WW development (YW).

$$\begin{array}{c} \text{Mathematically},\mathrm{DI}=\mathrm{YD}/\mathrm{YW}\\ \left(\text{Zhanget al}.,2007\right) \end{array}$$

### Statistical analysis

An analysis of variance (ANOVA) was performed by utilizing the general linear model (GLM) program to see the effects of various nitrogen levels, priming, and post-flowering drought stress on the physiological, morphological, and enzymatic exercises. The mean values for treatments were compared with the least significant difference (LSD *t*-test was applied to test the treatment differences.) with a probability of 0.05 (SPSS Inc., Chicago, IL, United States). The figures were drawn using Sigma Plot 10.

## Results

### Leaf water potential, osmotic potential, and leaf relative water content

**FIGURE 2:**
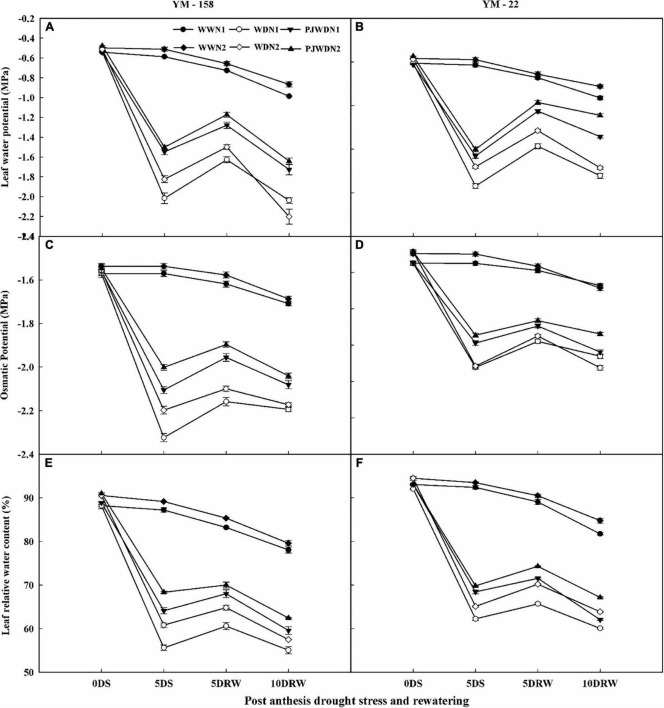
Effects of pre-drought priming on leaf water potential (Ψw), osmatic potential (Ψs), and leaf relative water content (LRWC) in response to post-anthesis drought stress under two nitrogen (N) rates (N1,180 and N2,300) in Yangmai-158 **(A,C,E)** and Yangmai-22 **(B,D,F)** wheat cultivars. Treatments: WW (control), no priming at jointing + no post-anthesis drought stress; PJWD, priming at jointing + post-anthesis drought stress; and WD, no priming at jointing + post-anthesis drought stress. Pre-drought priming was completed during the jointing stage, and post-anthesis drought stress was applied from 7 to 14 DAA. The horizontal axis shows the time course of the sampling: 1 day before drought stress (0DS), 5 days after drought stress (5DS), 5 and 10 days after re-watering (5DRW and 10DRW). Each vertical bar above the mean values indicates the standard error of three replicates (*n* = 3) by using three-way ANOVA at *P* < 0.05.

The leaf water potential (Ψw), osmotic potential (Ψs), and LRWC of flag leaves were measured at the post-anthesis stage and presented in Figures 2A–F. A decreasing trend of these parameters was observed from 12 to 14 days after anthesis (DAA) in both cultivars. The maximum value (less negative) of Ψw and Ψs was recorded in WW, PJWD, and WD under N2 supplementation. The Ψw, Ψs, and LRWC showed a gradual decrease in drought-stressed plants. Moreover, there was a significant difference in Ψw, Ψs, and LRWC in both N levels dose-dependently and WW, and there was also a significant difference between treatments under PJWD and WD conditions. The Ψw, Ψs, and LRWC values declined further under N1 compared to N2. The effect of drought stress decreased with higher nitrogen levels. For instance, N2 showed high performance as compared to N1 in WW, PJWD, and WD. Furthermore, under the post-anthesis drought stress, Ψw, Ψs, and LRWC were less reduced in primed plants when compared to the non-primed plants in both cultivars. After re-watering, the Ψw, Ψs, and LRWC recovered differently under different water and N levels. The drought-stressed plants showed better recovery in Ψw, Ψs, and LRWC under N2 when compared with N1.

### Gas exchange parameters

**FIGURE 3:**
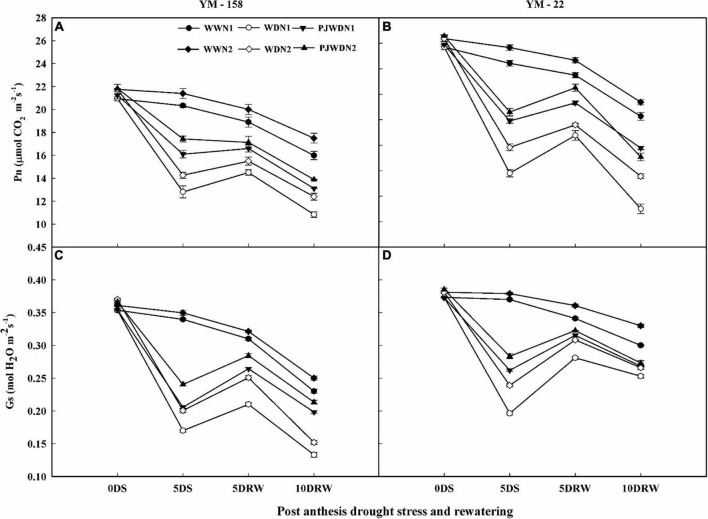
Effects of pre-drought priming on net photosynthetic rate (Pn) and stomatal conductance (gs) in response to post-anthesis drought stress under two nitrogen (N) rates (N1,180 and N2, 300) in Yangmai-158 **(A,C)** and Yangmai-22 **(B,D)** wheat cultivars. Treatments: WW (control), no priming at jointing + no post-anthesis drought stress; PJWD, priming at jointing + post-anthesis drought stress; and WD, no priming at jointing + post-anthesis drought stress. Pre-drought priming was completed during the jointing stage, and post-anthesis drought stress was applied from 7 to 14 days after anthesis. The horizontal axis shows the time course of the sampling: 1 day before drought stress (0DS), 5 days after drought stress (5DS), and 5 and 10 days after re-watering (5DRW and 10DRW). Each vertical bar above the mean value indicates the standard error of three replicates (*n* = 3) by using three-way ANOVA at *P* < 0.05.

The level of photosynthesis was maximum from 12 to 14 DAA in both cultivars, and then it started to decrease gradually after 14 days post-anthesis. Analysis showed that the photosynthesis rate (Pn) and stomatal conductance (gs) were reduced under post-anthesis WD and PJWD treatments relative to the WW treatment. However, under WD, the photosynthesis (Pn), and stomatal conductance (gs) were reduced relative to PJWD in YM-158 and YM-22 varieties. Lower Pn and gs were observed in WW, WD, and PJWD conditions under N1 compared to N2 (Figures 3A–D). The Pn and gs were influenced more under drought stress than priming treatments when compared to WW, depending on the N level. After re-watering, the PJWD plants showed higher recovery than the WD plants in both cultivars. Furthermore, slow recovery was observed under N1 compared to N2. The results indicated that higher nitrogen and priming have positive effects on Pn and gs under drought conditions.

### Chlorophyll fluorescence measurements

**FIGURE 4:**
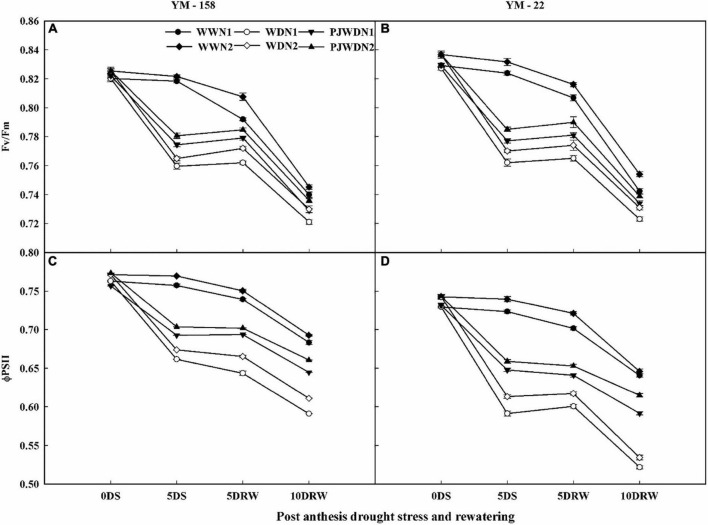
Effects of pre-drought priming on the quantum efficiency of the photochemical reaction in PSII (Fv/Fm) and the quantum yield of the PSII electron transport (ΦPSII), in response to post-anthesis drought stress under two nitrogen (N) rates (N1,180 and N2, 300) in Yangmai-158 **(A,C)** and Yangmai-22 **(B,D)** wheat cultivars. Treatments: WW (control), no priming at jointing + no post-anthesis drought stress; PJWD, priming at jointing + post-anthesis drought stress; and WD, no priming at jointing + post-anthesis drought stress. Pre-drought priming was completed during the jointing stage, and post-anthesis drought stress was applied from 7 to 14 days after anthesis. The horizontal axis shows the time course of the sampling: 1 day before drought stress (0DS), 5 days after of drought stress (5DS), and 5 and 10 days after re-watering (5DRW and 10DRW). Each vertical bar above the mean value indicates the standard error of three replicates (*n* = 3) by using three-way ANOVA at *P* < 0.05.

The decline in Fv/Fm and Φ_*PSII*_ values started from 12 to 14 DAA. There was a significant alteration in Fv/Fm and Φ_*PSII*_ rates in N1 and N2 under WW, WD, and PJWD conditions. Drought-stressed WD significantly decreased the Fv/Fm and Φ_*PSII*_ rates of flag leaves, but PJWD in both cultivars maintained higher Fv/Fm and Φ_*PSII*_ values than WD. On the other hand, N2 supplement plants showed better stability and reduced effects of drought stress on these parameters than N1 (Figures 4A–D). The Fv/Fm and Φ_*PSII*_ rates in drought-stressed plants also varied from the rates of WW under drought stress and N treatments. The decline in Fv/Fm and Φ_*PSII*_ was more prominent in WD under N1 compared to N2. After re-watering, the value of Fv/Fm and Φ_*PSII*_ increased. The primed plants showed higher recovery rates than the non-primed plants in both varieties, and N2 recovered quicker than N1.

### Chlorophyll and soluble protein content measurements

**FIGURE 5:**
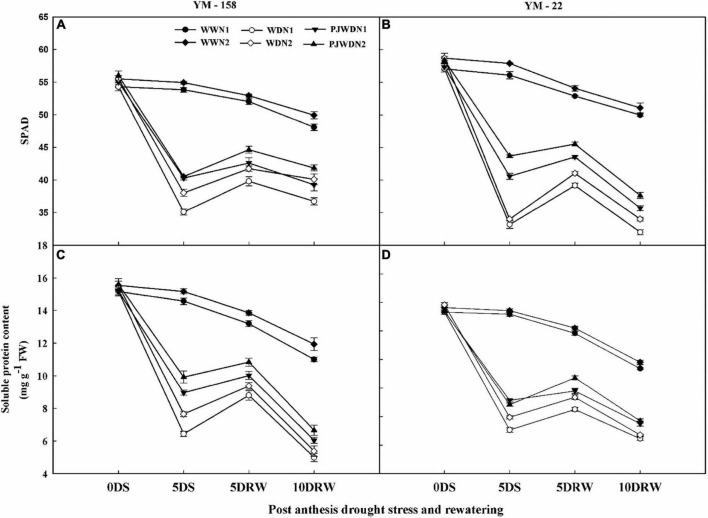
Effects of pre-drought priming on chlorophyll (SPAD value) and soluble protein content in response to post-anthesis drought stress under two nitrogen (N) rates (N1,180 and N2, 300) in Yangmai-158 **(A,C)** and Yangmai-22 **(B,D)** wheat cultivars. Treatments: WW (control), no priming at jointing + no post-anthesis drought stress; PJWD, priming at jointing + post-anthesis drought stress; and WD, no priming at jointing + post-anthesis drought stress. Pre-drought priming was completed during the jointing stage, and post-anthesis drought stress was applied from 7 to 14 days after anthesis. The horizontal axis shows the time course of the sampling: 1 days before drought stress (0DS), 5 days after drought stress (5DS), and 5 and 10 days after re-watering (5DRW and 10DRW). Each vertical bar above the mean value indicates the standard error of three replicates (*n* = 3) by using three-way ANOVA at *P* < 0.05.

The Chl and soluble protein content showed significant differences in all three water levels and N doses (Figures 5A–D). The content of Chl and soluble protein was significantly affected under the WD treatments depending on the strength of WD and N application doses. WD and PJWD significantly decreased the value of Chl and soluble protein in both wheat cultivars compared with WW. The plants under N1 showed a lesser value of Chl and soluble protein content compared to N2. Chlorophyll and soluble protein content under N2 showed more stability in WW, WD, and PJWD treatments compared to N1. After re-watering, the recovery of Chl and soluble protein was slower in N1 than in N2.

### Superoxide, hydrogen peroxide, and malondialdehyde content

**FIGURE 6:**
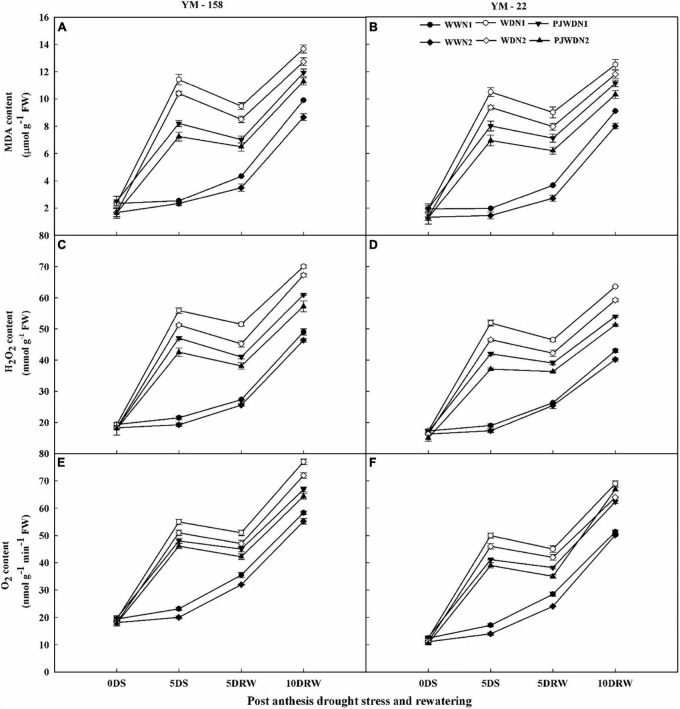
Effects of pre-drought priming on malondialdehyde (MDA), hydrogen peroxide (H_2_O_2_), and superoxide anion radical (O_2_^–^) content in response to post-anthesis drought stress under two nitrogen (N) rates (N1, 180 and N2, 300) in Yangmai-158 **(A,C,E)** and Yangmai-22 **(B,D,F)** wheat cultivars. Treatments: WW (control), no priming at jointing + no post-anthesis drought stress; PJWD, priming at jointing + post-anthesis drought stress; and WD, no priming at jointing + post-anthesis drought stress. Pre-drought priming was completed during the jointing stage, and post-anthesis drought stress was applied from 7 to 14 days after anthesis. The horizontal axis shows the time course of the sampling: 1 day before drought stress (0DS), 5 days after drought stress (5DS), and 5 and 10 days after re-watering (5DRW and 10DRW). Each vertical bar above the mean value indicates the standard error of three replicates (*n* = 3) by using three-way ANOVA at *P* < 0.05.

The O_2_^–^, H_2_O_2_, and MDA content increased rapidly in drought stress and in priming plants when compared to the WW plants at the post-anthesis stage. Significant differences in the concentrations of O_2_^–^, H_2_O_2_, and MDA were observed among the N levels in the WW, WD, and PJWD conditions dose-dependently. However, the concentration of these parameters decreased with increasing N levels in the WD and PJWD treatments. Therefore, in N2, the magnitude of O_2_^–^, H_2_O_2_, and MDA increment was less than N1 (Figures 6A–F). Compared to drought stress treatment, the primed plants showed less accumulation of O_2_^–^, H_2_O_2_, and MDA. Similarly, cultivar YM-158 showed higher content of O_2_^–^, H_2_O_2_, and MDA than that of YM-22, which suggested that YM-158 is more sensitive to drought than YM-22. After re-watering, the accumulation of O_2_^–^, H_2_O_2_, and MDA decreased, particularly in the primed and N2 plants, when compared with drought-stressed and N1-supplemented plants.

### Activities of anti-oxidative enzymes

**FIGURE 7:**
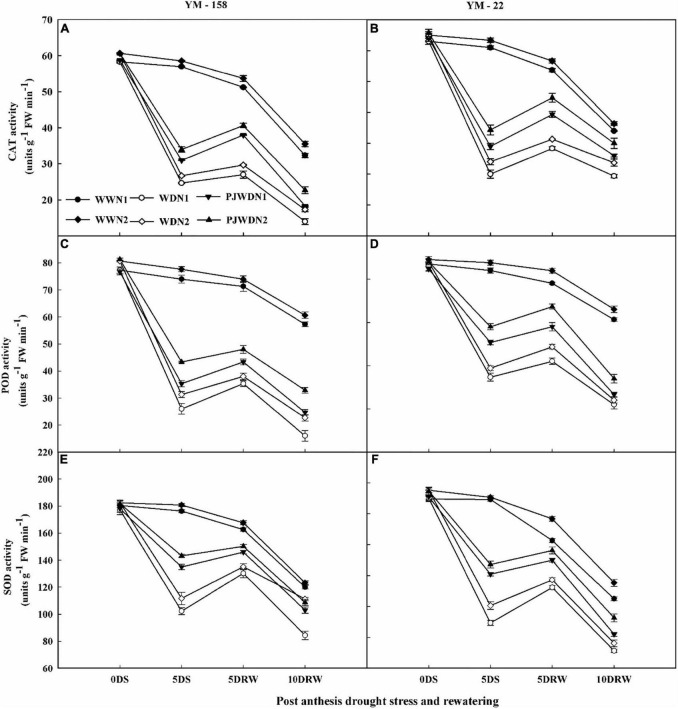
Effects of pre-drought priming on catalase (CAT), peroxidase (POD), and superoxide dismutase (SOD) content in response to post-anthesis drought stress under two nitrogen (N) rates (N1, 180 and N2, 300) in Yangmai-158 **(A,C,E)** and Yangmai-22 **(B,D,F)** wheat cultivars. Treatments: WW (control), no priming at jointing + no post-anthesis drought stress; PJWD, priming at jointing + post-anthesis drought stress; and WD, no priming at jointing + post-anthesis drought stress. Pre-drought priming was completed during the jointing stage, and post-anthesis drought stress was applied from 7 to 14 days after anthesis. The horizontal axis shows the time course of the sampling: 1 day before drought stress (0DS), 5 days after drought stress (5DS), and 5 and 10 days after re-watering (5DRW and 10DRW). Each vertical bar above the mean value indicates the standard error of three replicates (*n* = 3) by using three-way ANOVA at *P* < 0.05.

The POD, SOD, and CAT activities were significantly reduced under drought stress and priming treatments compared to the WW treatment (Figures 7A–F). There were significant differences in SOD, POD, and CAT activities between different N doses and water levels. In the WD condition, the POD, SOD, and CAT showed significant differences. In contrast, nitrogen treatment dose-dependently increased the activities of SOD, POD, and CAT in primed plants under drought stress treatment. After re-watering, the N2 plants recovered quickly when compared to N1 plants. The maximum value of all of these parameters was observed in the control treatment with N2. Under post-anthesis drought stress, the SOD, POD, and CAT activities slightly declined in primed plants when compared to the non-primed plants in both cultivars.

### Flag leaf area and specific leaf weight

**FIGURE 8:**
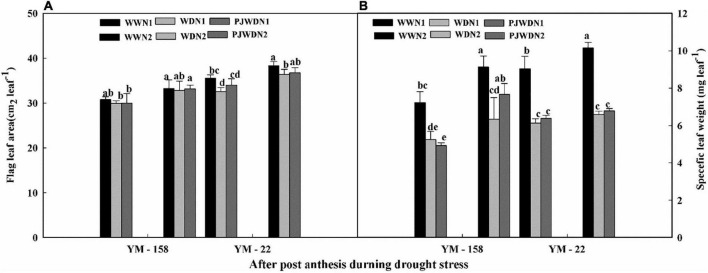
Effects of pre-drought priming on flag leaf area and specific leaf weight in response to post-anthesis drought stress under two nitrogen (N) rates (N1, 180 and N2, 300) in Yangmai-158 and Yangmai-22 **(A)**, and Yangmai-158 and Yangmai-22 **(B)** wheat cultivars. Treatments: WW, (control) no priming at jointing + no post-anthesis drought stress; PJWD, priming at jointing + post-anthesis drought stress; and WD, no priming at jointing + post-anthesis drought stress. Pre-drought priming was completed during the jointing stage, and post-anthesis drought stress was applied from 7 to 14 days after anthesis. Each vertical bar above the mean value indicates the standard error of three replicates (*n* = 3) by using three-way ANOVA at *P* < 0.05.

In the post-flowering stage, during the drought stress period, the flag leaf area and specific leaf weight of YM-22 were higher than those of YM-158 under WW, WD, and PJWD conditions (Figures 8A,B). The drought stress decreased flag leaf area and weight, while nitrogen and priming improved the flag leaf area and weight. The minimum flag leaf area and weight were observed in N1 under the WD condition. The maximum flag leaf area was recorded in WW with N2 in both cultivars. PJWD enhanced the flag leaf area and specific leaf weight more than WD. These outcomes showed that pre-anthesis PJWD treatment could bring a superior change in leaf morphology under post-anthesis WD conditions, supporting the accumulation of photosynthetic products.

### Grain filling duration, grain yield characteristics, and related parameters

The drought stress treatment produced significant changes in GFD, spikes pot –1, grains spike^–1^, spike length, biomass pot^–1^, thousand-grain weight, and grain yield pot ^–1^ (Table 2). However, drought priming lessened the loss caused by post-anthesis drought stress. Low N aggravated the effects of drought stress on these attributes. The level of grain yield reduction increased with increasing drought and N insufficiency. Drought-primed plants indicated less reduction in grain yield when compared to the non-primed plants in both cultivars under normal water conditions. Similarly, the grain yield reduced less under N2, which resulted in a more prominent drought index (DI) for drought stress treatments. Considerably higher DI was noted in N2 (PJWD) and lower DI was seen in N1 (WD). The impacts of drought stress treatments and N amount on grain yield were assigned to their remarkably similar effects on spikes pot ^–1^, grains spike^–1^, spike length, biomass pot^–1^, and thousand-grain weight under N1 drought stress conditions. The water level and N showed significant effects on the spikes pot^–1^. N2 showed improved results compared to N1. Under WW, the number of grains spike^–1^ was better than WD and PJWD, whereas the number of grains spike^–1^ increased with an increasing level of nitrogen. Spike length significantly decreased as a response to PJWD and WD, and nitrogen content also affected the spike length. The thousand-grain weight was also influenced by drought stress and nitrogen level. However, the response to nitrogen was different than the water level and increased the thousand-grain weight. Furthermore, biomass at maturity decreased significantly due to post-anthesis drought stress and priming when compared to WW treatment, and its reduction was also greater in N1 compared to N2. Therefore, it can be said that a high dose of nitrogen N2 and priming before anthesis can reduce the impact of post-anthesis drought stress and increase drought resistance in plants.

**TABLE 2:** Effects of pre-drought priming and nitrogen on grain yield and its components under post-anthesis drought stress in Yangmai-158 (YM-158) and Yangmai-22 (YM-22) wheat cultivars.

Treatment			Grain filling duration (d)	Spikes pot^–1^	Grains spike^–1^	Spike length (cm)	Biomass pot^–1^(g)	1,000- grain weight (g)	Grain yield pot^–1^(g)	Drought index
YM − 158	N1	WW	38.1abc	25.48cd	36.91fg	7.31cd	120.3bcde	37.4cd	42.0ef	0e
		WD	34.7e	24.19d	31.723i	5.18f	108.0ef	32.5e	35.5i	0.84d
		PJWD	35de	24.81cd	35.8gh	7.46cd	113.33de	34.1de	37.3hi	0.89bc
	N2	WW	38.4ab	29.487a	43.02b	9.58a	121.3bcde	43.4ab	48.1b	0e
		WD	36de	28.52ab	40.09de	6.88de	111.3de	36.13de	43.6de	0.91b
		PJWD	36.8bcd	28.84a	42.45bc	9.21ab	120.6bcde	41.8ab	45.7cd	0.95a
YM − 22	N1	WW	39.5a	26.47bc	38.87def	7.81bcd	124.0abcd	40.1bc	44.2cde	0e
		WD	35.8de	25.50cd	33.75hi	5.53ef	114.0de	34.1de	38.4gh	0.86cd
		PJWD	37.2bcd	26.067cd	37.93efg	7.43cd	117.3cde	36.6d	39.8fg	0.9bc
	N2	WW	39.0a	30.16a	45.44a	9.66a	135.0a	44.65a	50.6a	0e
		WD	36.9bcd	29.233a	42.03c	6.90de	127.3abc	41.5ab	46.3bc	0.91ab
		PJWD	38.9a	29.767a	43.81ab	9.14abc	131.3ab	43.5ab	48.2b	0.95a
**Probability level of ANOVA**										
Factors										
N			**	**	**	**	**	**	**	**
WL			**	**	**	**	**	**	**	**
V			**	**	**	ns	**	**	**	ns
N*WL*V			**	ns	ns	ns	ns	ns	ns	ns

ns, non-significant and “*”, “**”, significant difference at 0.05 and 0.01 levels of probability, respectively. The different lowercase letters within the table showed significant differences at p-value 0.05. N, Nitrogen; WL, water level; V, variety.

## Discussion

The ability of the wheat plant to counteract drought stress in the post-anthesis stage varied with the variation in its treatments with priming and N level (Abid et al., 2016a). The results of the present study showed that drought stress reduced plant growth, while higher N application and pre-anthesis moderate drought stress (termed as priming) eased the negative effects of drought stress. In addition, under drought stress, stomatal conductance was decreased, which led to reduced photosynthesis when compared with other treatments. LRWC is the main characteristic that shows plant water level, and when LRWC drops, it causes stomatal closure and affects the availability of CO_2_ during photosynthesis under water deficit conditions (Moshelion et al., 2015). In this study, higher Ψw, Ψs, and LRWC values were observed under higher N application and pre-anthesis drought priming in response to drought stress when compared with non-primed plants, indicating that priming and N application trigger leaf activity that enables plants to continue normal functioning under drought stress conditions and helps plants to recover quickly once the drought is over, as demonstrated by the re-watering of the primed plants. Wang et al. (2014a,b) observed higher LRWC and stomatal conductance in the primed plants than in non-primed plants under drought stress conditions in wheat. Similarly, higher N application increased the stomatal conductance and leaf characteristics relative to low N application in drought-stressed plants, suggesting that under low N conditions, the leaf water potential would be decreased, which limits cell growth. As previously mentioned, reduced Ψw under abiotic stresses is the major source of decreased accumulation of dry matter and grain yield (Gepstein and Glick, 2013). Our result showed that under drought conditions, the osmotic potential and LRWC in primed plants in both varieties were higher than those in the non-primed plants. These findings confirmed that drought-primed plants could maintain good water status by better controlling the osmotic potential to cope with drought stress after flowering.

In this study, the decrease in Chl content under drought stress might be the reason for the decline of the photochemical activity of chloroplast, which ultimately reduces photosynthetic activity (Moshelion et al., 2015). However, compared with the non-primed plants and N1 supplementation, the primed and N2-supplemented plants restored the photochemical activities to some extent. It is worth noting that the obvious effect of limited N supply on the droughted plants is the decrease of the Chl and Rubisco contents (Abid et al., 2016a). As we know from the previous research, the larger part of N is stored in enzymes, such as Rubisco (which is the main source of the N recycling), and is a necessary component of enzymes involved in the photosynthetic process (Abid et al., 2016a). Previously, similar effects of nitrogen limitation on Rubisco concentration and photosynthesis were pointed out in rose plants by Gonzalez-Real and Baille (2000), as well as in rice and wheat plants (Makino, 2011**)**. Under severe drought stress, the carboxylation efficiency of Rubisco was incredibly reduced, and Rubisco acts as an oxygenase rather than a carboxylase, where it can reduce the carbon assimilation of photosynthesis (Makino, 2011). N is the main part of Chl and proteins, influencing the entire metabolism of plants under drought stress. Grassi and Magnani, (2005) found that adequate N content partially restored the photosynthetic biochemistry compared to limited N availability. Previous studies have reported that when the drought stress level intensifies, it reduces the leaf area, which subsequently affects photosynthetic pigmentation and reduces stomatal conductance and transpiration rate (Ahmad et al., 2022). In this manner, supporting the plant’s water status and stomatal opening under high N is important to maintaining the conductance of leaf to CO_2_, photosynthetic responses, and electron transfer (Liu et al., 2013).

MDA is the main product of lipid peroxidation, which reflects the degree of damage to the plant photosynthetic functions under adverse conditions (Zeeshan et al., 2020, 2021). Severe drought stress under low nitrogen application causes excessive accumulation of MDA. The excessive accumulation of MDA triggers early leaf senescence, causes degradation of chlorophyll and macromolecules, and increases cell membrane permeability, which eventually largely affect the development of grains (Zeeshan et al., 2020). We reported higher MDA and ROS accumulation in response to N1-supplemented and droughted plants (DS) when compared with N2-supplemented and primed plants (PJWD) under the same conditions. The strict control of ROS and MDA production and accumulation is to plant defense mechanisms and is a necessary phenomenon under unfavorable conditions because the excessive accumulation of these cellular toxicants can cause cell death, thereby decreasing plant growth and development (Bieker and Zentgraf, 2013).

In order to cope with oxidative damage and keep a balance between the production and scavenging of reactive oxygen species and restoration of metabolic homeostasis, plants have to evolve a well-established antioxidant enzyme system (Najafi et al., 2021; Salam et al., 2022). A study has found a strong correlation between oxidative stress caused by drought and the activities of antioxidant enzymes (Adrees et al., 2020). The antioxidative enzyme system of plants, mainly comprising SOD, POD, and CAT, has a prominent role in the mitigation of oxidative stress (Szalai et al., 2009). Our study reported elevated activity of SOD, POD, and CAT in flag leaves in response to the PJWD and higher application of N (N2) over non-primed plants supplemented with limited N (N1). These results suggest that the observed increase in SOD and POD activities would compensate for the accumulation of ROS. In addition, the increase in CAT activity would lead to scavenging the H_2_O_2_, thereby reducing oxidative damage to the membrane. SOD is a key enzyme for the scavenging of reactive oxygen species and conversion of O_2_− to H_2_O_2_. Finally, CAT and POD subsequently scavenge the H_2_O_2_ produced due to dismutation and convert it into H_2_O and O_2_ using ascorbate in the process (Khan et al., 2020). This study recommends that priming and high doses of N can increase the antioxidant capacity after flowering to reduce the damage caused due to oxidative stress, which can be advantageous in improving the physiological activity of plant leaves. Under higher nitrogen application, the higher ROS detoxification by antioxidant systems in drought-stressed plants may help in protecting photosynthesis. Wang et al. (2014a,b) found higher antioxidant activities and lower accumulation of ROS and MDA in pre-stress primed plants over the non-primed plants under droughted conditions in wheat at different growth stages. Furthermore, Saneoka et al. (2004) observed that N nutrition promotes the drought tolerance of bent grass by stopping cell membrane damage, reducing MDA accumulation, and improving osmoregulation in Agrostis palustris Huds.

Under the droughted condition, crops alter their phenophases: they trigger early anthesis and early maturity in order to avoid stressful conditions, and this alteration is considered vital for maintaining higher grain yield, particularly in cereal (Chaves et al., 2002). This alteration could shift the post-anthesis stage for suitable conditions for leaf photosynthesis and grain filling in wheat (Fan et al., 2017). In the current study, GFD was reduced under the WD condition relative to other treatments, which in turn decreased the grain weight. Previously, Lv et al. (2021) mentioned that in wheat crops, both grain filling rate (GFR) and GFD have a direct impact on grain weight. Similarly, Wu et al. (2011) observed that during the vegetative growth stage, the drought stress alone and/or limited N supply significantly reduced the grain weight and hindered the yield. In the current study, upon supplementation with high N treatment (N2), plants produced more grains and increased plant green canopy, resulting in elevated photosynthetic activity and a high carbon accumulation. On the other hand, droughted plants (WD) under low nitrogen supplementation produced fewer grains and a smaller canopy, resulting in decreased photosynthetic activity. According to an estimation, about 30–50% of the photosynthetic products required for wheat grain filling are delivered through the current photosynthesis during grain filling, so the “keep green” feature can extend the duration of its availability (Inoue et al., 2004; Abid et al., 2016c). In addition, the ability of the PJWD plants to maintain a higher photosynthetic rate and chlorophyll content may be the ultimate cause for the increase in grain filling rate and duration, and ultimately higher grain yield as compared to the WD plants. The grain filling rate and duration are the key factors that determine the final grain weight, and the final grain weight is crucial for final grain yield (Altenbach, 2012). Therefore, compared with WD plants at post-anthesis, the PJWD plants maintained a higher sink strength, had satisfactory translocation, and received grain reserves, confirming the successful development of grains, and limiting their severe dry weight and grain yield decline.

Furthermore, the PJWD plants efficiently prevented water loss in grains under drought conditions, which might avoid the vascular physiochemical gap within the grain. As the endosperm and embryo dehydration could limit the metabolism of obtained assimilates, the hydraulic response of the developing grain to drought could have a direct impact on filling quality (Egli and TeKrony, 1997; Gupta et al., 2011). Under unfavorable environmental conditions, particularly at the post-anthesis stage, plant physiological activities can be reduced and affect the grain yield. Therefore, to face the unfavorable conditions and reduce yield losses, the application of N and priming are needed before post-anthesis drought stress. A higher application of N (N2) along with PJWD plants produced notably higher grain yield and yield-related parameters when compared to remaining treatments except WW. Taken together, the tandem application of higher N with pre-anthesis drought priming can reduce the drought stress on the wheat plants at the post-anthesis stage and can improve the yield of wheat in response to drought stress.

## Conclusion

The results of this study indicated that wheat plants pre-exposed to moderate drought stress (priming) and an adequate supply of nitrogen have reduced adverse effects of drought stress after anthesis. Nitrogen and priming had significant effects on wheat’s physiological activities and grain yield. The study found that high nitrogen and priming before anthesis induced greater photosynthetic capacity by expanding the green flag leaf area, elevating the chlorophyll and soluble protein content, increasing sink strength, and increasing the GFD, thereby increasing the grain weight and yield. Furthermore, high nitrogen and priming also increased antioxidant capacity and reduced oxidative stress and MDA accumulation. Drought priming in the early growth stages (especially during the jointing period) highlighted the importance of drought timing and was confirmed to be the best approach to trigger the plants to initiate an effective tolerance mechanism against drought stress. Agriculture will face severe water shortage in the future, and hence to face the challenge of water scarcity, we should not only rely on breeding new cultivars that can tolerate water scarcity, but also invent crop management techniques that can increase crop potential to achieve sustainable crop production.

## Data availability statement

The original contributions presented in this study are included in the article/supplementary material, further inquiries can be directed to the corresponding author.

## Author contributions

AU, ZT, and TD conceived and organized the experiment. AU conducted the experiment, collected the samples, analyzed the data, and wrote the draft. LX, KL, CS, AK, and YJ helped in plant sampling and data analysis. MA and MZ critically revised the manuscript. TD acquired the funding. All authors contributed to the article and approved the submitted version.
